# Early Transcriptional Changes in Feline Herpesvirus-1-Infected Crandell-Rees Feline Kidney Cells

**DOI:** 10.3390/vetsci11110529

**Published:** 2024-10-30

**Authors:** Xiuqing Xiao, Fuqiang Xu, Fan Jia

**Affiliations:** 1 Shenzhen Key Laboratory of Viral Vectors for Biomedicine, Shenzhen-Hong Kong Institute of Brain Science, Shenzhen Institute of Advanced Technology, Chinese Academy of Sciences, Shenzhen 518055, China; xiaoxiuqing24@mails.ucas.ac.cn (X.X.); fq.xu@siat.ac.cn (F.X.); 2 Key Laboratory of Quality Control Technology for Virus-Based Therapeutics, The Brain Cognition and Brain Disease Institute, Shenzhen Institute of Advanced Technology, Chinese Academy of Sciences, Shenzhen 518055, China; 3 University of Chinese Academy of Sciences, Beijing 100049, China; 4 Faculty of Life and Health Sciences, Shenzhen University of Advanced Technology, Shenzhen 518107, China

**Keywords:** FHV-1, transcriptome, early post-infection, immune responses, virus–host interactions

## Abstract

FHV-1 is a highly contagious pathogen that significantly threatens feline health and contributes to rising pet healthcare costs. The mechanisms underlying FHV-1 and host interactions remain poorly understood. For the first time, we conducted a systematic analysis of transcriptomic changes in CRFK cells following FHV-1 infection using RNA-seq. The differentially expressed genes (DEGs) displayed significant associations with cellular components, particularly the chromatin structure. Pathway analysis of the DEGs highlighted key host immune responses, including Toll-like receptors (TLRs), IL-17, TNF, MAPK, and Rap1 signaling pathways. By integrating the RNA-seq and RT-qPCR results, we identified CXCL8, CXCL10, MMP1, MMP9, CSF2, CSF3, CCL20, TLR2, TLR3, TLR4, TNF, and FOS as potentially important genes in the host’s immune response to FHV-1. These findings provide valuable insights into the mechanisms underlying FHV-1 and host interactions.

## Simple Summary

Feline herpesvirus-1 (FHV-1) is a major cause of infectious diseases in cats, leading to respiratory and eye infections. In this study, we investigated the early genetic changes that occur in Crandell-Rees Feline Kidney (CRFK) cells after being infected with FHV-1. Using RNA sequencing (RNA-seq) techniques, we analyzed the molecular interactions between the virus and the host cells. Our results identified important immune response genes that become active shortly after infection, highlighting pathways related to FHV-1 and host interactions. These findings provide a better understanding of the early stages of FHV-1 infection and offer a foundation for future research aimed at improving the diagnosis and treatment.

## 1. Introduction

FHV-1 is a main pathogen causing viral rhinotracheitis, pneumonia, and ocular disease in cats. Kittens are usually infected with FHV-1 after 6–9 weeks of birth due to a decline in maternal antibodies. Infected cats exhibit neurological symptoms, fever, pneumonia, ocular lesions, and a high fatality rate due to the loss of passive immunity [1,2,3]. Serological studies have estimated that up to 97% of cats display seropositivity for FHV-1, and over 80% of these infected cats remain persistently infected for their entire lives [4,5]. After the acute infection phase, as with other α-herpesviruses, FHV-1 establishes latency in the trigeminal ganglia. Stress or immunosuppression can reactivate the virus, resulting in the shedding of infectious particles and the recurrence of clinical signs [6]. Commercially available modified live vaccines containing FHV-1 are generally safe and effective. However, the current vaccines have limitations in terms of duration and efficacy, as they do not prevent infection, viral nasal shedding, or latency [7]. Recent research has focused on designing candidate vaccines to control FHV-1 infection in cats, targeting the pathogenesis and immunogenicity of FHV-1 [8,9,10,11,12]. It is known that FHV-1 enters cells through receptor-mediated endocytosis, a process that depends on both pH and dynamin [13]. The primary mechanisms of cell death induced by FHV-1 infection include apoptosis, loss of cell-to-cell contact, cell rounding, and detachment. Infected cells predominantly undergo apoptosis and cell death due to disrupted intercellular contact [14]. FHV-1 triggers apoptosis and autophagy in CRFK cells in a time- and dose-dependent manner, and there is crosstalk between FHV-1-induced autophagy and apoptosis [15,16]. FHV-1 inhibits the host type I interferons (IFNs) pathway by blocking IRF3 dimerization via its US3 protein. This strategy allows the virus to evade the host innate immune response effectively, establishing a latent infection in cats [17]. Despite existing research providing insights into FHV-1 pathogenesis, mechanisms underlying the interactions between FHV-1 and host cells remain insufficiently understood. Therefore, it is crucial to deepen our understanding of FHV-1 infection and pathogenesis.

Analyzing host transcriptome changes and immune responses can deepen our understanding of the mechanisms driving the host response to FHV-1 infection. RNA-seq has become essential for elucidating gene expression dynamics and has demonstrated early transcriptome differences in host cells following serval viral infections. A transcriptome analysis of rabies virus revealed that early differential gene expression significantly influences neuronal function through pathways such as IL-17 and MAPK, highlighting the importance of early-stage interventions to alleviate viral pathogenicity [18]. Comparative transcriptome data from attenuated and wild-type rabies viruses indicated that the attenuated strain reduces the activation of pattern recognition receptors (PRRs) like RIG-I-like receptors (RLRs) and Toll-like receptors (TLRs), as well as their downstream pro-inflammatory pathways (e.g, IRF1, 5, 7, NFkB1, 2, IFNAR, and IFNGR), thereby decreasing cytotoxicity [19]. Feline coronavirus (FCoV) infection has been shown to positively regulate the MAPK signaling pathway and downregulate T-cell-related processes, affecting host immune homeostasis [20,21]. Based on previous studies on transcriptome results, RNA-seq has proven to be a crucial tool for understanding virus–host interactions and host immune responses. Until now, no transcriptomic studies on FHV-1 infections have been available.

In this study, we systematically analyzed the alterations in the host’s overall transcriptome caused by FHV-1 infection using RNA-seq for the first time. The early transcriptome changes in FHV-1 infected CRFK cells reflects alterations in the host’s immune responses, providing new insights into FHV-1 and host interactions.

## 2. Materials and Methods

### 2.1. Cells and Viruses

The CRFK cells (CCL-94, ATCC, Manassas, VA, USA) were cultured in DMEM (Gibco, Thermo Fisher, Waltham, MA, USA) supplemented with 10% fetal bovine serum (Gibco, Thermo Fisher, Waltham, MA, USA) at 37 °C in 5% CO_2_. The wild-type FHV-1 (Feline herpesvirus 1 strain GD2019, GenBank accession no. PP942287) was cultured in CRFK cells.

### 2.2. Plaque Assay

To illustrate the growth kinetics of FHV-1 in CRFK cells, plaque assay was performed to test the viral titer using the previous method [22]. Briefly, viral samples were serially diluted in 10-fold increments, and 100 μL of the diluted samples was transferred to individual wells of 6-well plates seeded with CRFK cells. These plates were incubated for 1 h at 37 °C in a 5% CO_2_ atmosphere to allow for viral adsorption. Subsequently, a first layer of 1.2% agar was added. After 48 hpi, a second layer of 1.2% agar layer containing neutral red was applied. Plaques were enumerated following an additional 24 h incubation period, and viral titers were expressed as plaque-forming units (PFUs) per milliliter.

### 2.3. Sample Collection and RNA Extraction

CRFK cells were initially seeded into T25 flasks and cultured in DMEM supplemented with 10% fetal bovine serum. After hours of growth, the cells were exposed to FHV-1 at a multiplicity of infection (MOI) of 0.05, followed by a 1 h incubation at 37 °C in a 5% CO_2_ environment. Post-incubation, cells were washed three times with PBS and were subsequently maintained in DMEM containing 2% fetal bovine serum. Samples of infected and uninfected cells were collected at 0, 3, and 6 hpi as indicated by the FHV-1 growth profiles. Five biological replicates were used to ensure accuracy.

RNA extraction was carried out using TRIzol^®^ Reagent (Invitrogen, Carlsbad, CA, USA), adhering to the provided protocol. The quality and integrity of the RNA were evaluated with an Agilent 2100 Bioanalyzer (Agilent Technologies, Santa Clara, CA, USA) alongside agarose gel electrophoresis, while concentration measurements were performed using a NanoDrop 2000 spectrophotometer (NanoDrop Technologies, Wilmington, DE, USA). Only samples with an OD260/280 ratio of 1.8–2.2, OD260/230 ≥ 2.0, an RNA integrity number (RIN) of at least 8, and a concentration of 50 ng/μL or higher were selected for further processing.

### 2.4. Library Construction and RNA-seq

For RNA sample preparation, total RNA served as the initial input. Libraries were constructed using the Fast RNA-seq Lib Prep Kit V2 (ABclonal, Wuhan, China). In brief, mRNA was isolated from the total RNA through poly-T oligo magnetic bead binding. Fragmentation occurred in the presence of divalent cations at elevated temperatures within the First Strand Synthesis Reaction Buffer (5X). The first strand of cDNA was synthesized with random hexamer primers and M-MuLV Reverse Transcriptase (RNase H-), followed by second-strand cDNA synthesis using DNA Polymerase I and RNase H. Blunt ends were achieved via exonuclease and polymerase activities. After adenylation at the 3′ ends, adaptors containing hairpin loops were ligated for hybridization readiness. To ensure selection of cDNA fragments sized 370–420 bp, AMPure XP system purification was applied. PCR amplification was carried out using Phusion High-Fidelity DNA polymerase, along with universal and Index (X) primers. The Agilent Bioanalyzer 2100 system (Agilent Technologies, Santa Clara, CA, USA) was used to assess library quality. Sample clustering was then conducted on the cBot Cluster Generation System with the TruSeq PE Cluster Kit v3-cBot-HS (Illumina, San Diego, CA, USA), as instructed by the manufacturer. Sequencing was performed on an Illumina Novaseq 6000, producing paired-end reads of 150 bp.

### 2.5. Quality Control and Read Mapping

Raw sequencing data in fastq format underwent initial processing with sickle (version 1.2). This step involved removing reads containing adapters, sequences with poly-N, and low-quality bases to produce clean data. The quality metrics, including Q20, Q30, and GC content, were calculated for the cleaned data, which formed the basis for all subsequent analyses. The reference genome and gene annotation files were sourced directly from the genome database. Hisat2 (v2.0.5) (https://daehwankimlab.github.io/hisat2/, accessed on 24 May 2024) was used to build the reference genome index, and paired-end reads were aligned to the genome using the same tool. Hisat2 was chosen for its ability to generate splice junction databases based on gene annotations, providing superior alignment performance compared to other tools.

### 2.6. Differential Expression Analysis

Differential expression analysis between infected groups and control groups, each with 5 biological replicates, was conducted using the DESeq2 R package (version 1.20.0). DESeq2 offers statistical methods to identify differential expression in digital gene expression data, based on the negative binomial distribution. *p*-values were adjusted for multiple testing using the Benjamini–Hochberg method to control the false discovery rate. Genes with an adjusted *p*-value (padj) of ≤ 0.05 identified by DESeq2 and a value of |log2(FoldChange)| ≥1 were considered as DEGs compared to the non-infected condition.

### 2.7. Gene Ontology (GO) and KEGG Enrichment Analysis

Transcripts and corresponding genes were annotated using the GO (http://www.geneontology.org, accessed on 24 May 2024) and Kyoto Encyclopedia of Genes and Genomes (KEGG, http://www.genome.jp/kegg/, accessed on 24 May 2024) databases. GO enrichment analysis of DEGs was conducted using the clusterProfiler R package, which corrected for gene length bias. GO terms with a corrected *p*-value (padj) ≤ 0.05 were deemed significantly enriched among the DEGs. The KEGG database serves to elucidate high-level biological functions—from molecular details to large-scale genomic datasets. The statistical enrichment of DEGs in the KEGG pathways was assessed using the clusterProfiler R package 3.16.1. KEGG pathways with padj ≤ 0.05 were regarded as enriched.

### 2.8. Validation of the RNA-Seq Results

RT-qPCR was conducted to validate the DEGs identified from RNA-seq. The total RNA was extracted using TRIzol^®^ Reagent (Invitrogen, Carlsbad, CA, USA) according to the manufacturer’s instructions. cDNA synthesis was performed with the TransScript One-Step gDNA Removal and cDNA Synthesis SuperMix (TransGen Biotech, Beijing, China). RT-qPCR reactions (20 μL total volume) consisted of 10 μL SYBR Green PCR kit (BioRad, Hercules, CA, USA), 0.8 μL primers (forward and reverse, 10 μM), 2 μL cDNA, and 6.4 μL ddH_2_O. The thermal profile was 95 °C for 30 s, then 40 cycles of 95 °C for 5 s and 60 °C for 30 s. Specific amplification was confirmed by melting curve analysis. RT-qPCR was performed in a 96-well plate on an ABI QuantStudio 3 system (Applied Biosystems, Carlsbad, CA, USA). Viral RNA levels were measured using glycoprotein B (gB) gene-specific primers for FHV-1, with the RPS7 gene as the reference. Among the housekeeping genes tested, RPS7 was the most stable in CRFK cells, outperforming GAPDH (Supplementary Figure S1), which is why we used RPS7 in our calculations [23]. Gene expression levels were quantified using the 2^−ΔΔCt^ method [24]. *t*-tests were used to assess significant differences. Correlation analysis was conducted with GraphPad software version 6.0 (San Diego, CA, USA).

## 3. Results

### 3.1. Global Transcriptome Changes Induced by FHV-1 Infection in CRFK Cells

**Figure 1:**
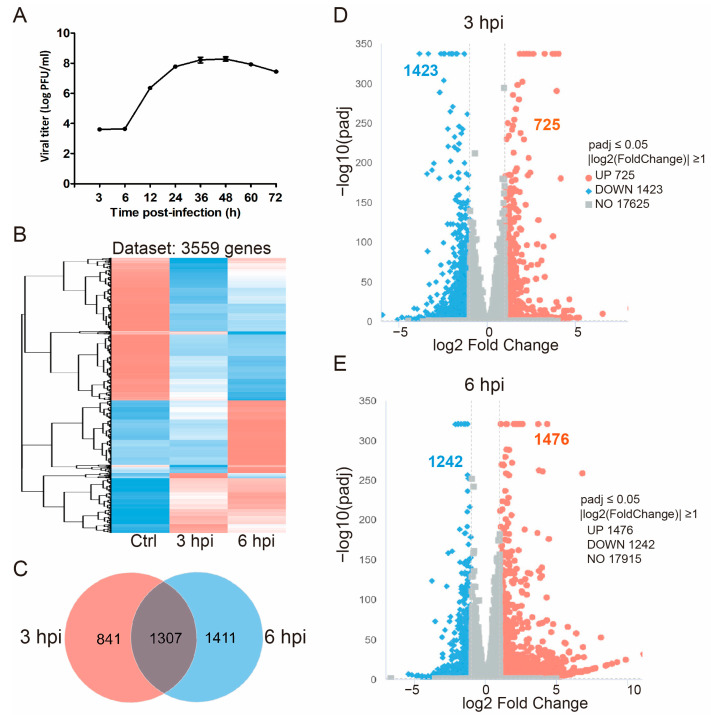
RNA-seq to analyze global transcriptome changes at 3 and 6 hpi. (**A**) Plaque assay of FHV-1 in CRFK cells, with each data point representing the average of three biological replicates. The error bar indicates standard deviation. (**B**) Heat map analysis classifying gene expression patterns at 3 and 6 hpi, each with 5 biological replicates. Genes with similar expression patterns are clustered. Red indicates high expression levels, and blue indicates low expression levels. (**C**) Venn diagram displaying the numbers of DEGs for each comparison. (**D**) Volcano plot of DEGs at 3 hpi. (**E**) Volcano plot of DEGs at 6 hpi. Red points indicate upregulated genes, blue points indicate downregulated genes, and grey points represent genes without significant changes.

To determine the suitable time for collecting the cell samples to analyze the early transcriptome alteration in cells after FHV-1 infection, the CRFK cells were infected by FHV-1 at an MOI of 0.05, and the supernatant was collected at 3, 6, 12, 24, 36, 48, 60, and 72 hpi. We determined the growth curves by testing the viral titer of each sample at the indicated time points by using a plaque assay. The viral titer was increased with time course from 3 to 48 hpi (Figure 1A). Therefore, the FHV-1 infected CRFK cells were collected at 3 and 6 hpi to perform the RNA-seq. To identify the DEGs, the samples from 3 and 6 hpi were compared to the control samples. Using the Illumina HiSeq platform, a total of 694 million raw reads were generated. Low-quality reads were filtered out, resulting in 675.92 million clean reads (see Supplementary Table S2). The heatmap revealed a total of 3559 DEGs (Figure 1B). Further analysis using Venn diagrams categorized these DEGs into three major groups (Figure 1C). Specifically, 841 and 1411 DEGs were uniquely expressed at 3 and 6 hpi in the FHV-1-infected CRFK cells, respectively, compared to the control sample. Additionally, 1307 DEGs were co-expressed at both 3 and 6 hpi compared to the control sample. A comprehensive list of all DEGs at 3 hpi compared to the control sample is provided in Supplementary Table S3, and a similar list for 6 hpi is available in Supplementary Table S4. Genes expressed exclusively at 3 hpi compared to the control group are detailed in Supplementary Table S5, while those expressed only at 6 hpi are listed in Supplementary Table S6. The expression levels of these DEGs significantly differed from the control group at both 3 and 6 hpi, with padj < 0.05. As illustrated in the volcano plot (Figure 1D,E), 1423 genes were downregulated and 725 genes were upregulated at 3 hpi, while 1242 genes were downregulated and 1476 genes were upregulated at 6 hpi in CRFK cells. These DEGs are associated with immune responses and may play a crucial role in the host’s immune response to FHV-1 infection.

### 3.2. Annotation of DEGs Based on GO Analysis

**Figure 2:**
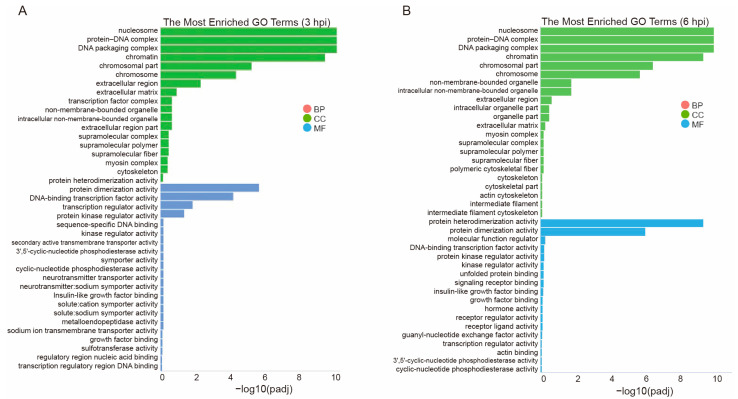
Top 40 GO terms of DEGs expressed at 3 and 6 hpi. GO terms were categorized into three groups: cellular component (CC), molecular function (MF), and biological process (BP). The top 40 GO terms were selected based on padj value. (**A**) GO annotation of DEGs expressed at 3 hpi. (**B**) GO annotation of DEGs expressed at 6 hpi.

To understand the early regulatory processes during FHV-1 infection, a further analysis focusing on GO terms was performed. GO terms with padj values < 0.05 were considered significantly enriched. The enriched GO terms were categorized into biological processes (BPs), cellular components (CCs), and molecular functions (MFs). The top 40 GO terms were selected based on the padj value, revealing that the GO analysis and enrichment results were consistent for both transcripts (Figure 2). The top three significant GO terms expressed by DEGs in both the 3 and 6 hpi groups were the nucleosome (GO:0000786), protein–DNA complex (GO:0032993), and DNA packaging complex (GO:0044815), all of which fall under the CC category. Most DEGs at 3 and 6 hpi were associated with host chromatin structure-related cellular components, and signal transduction-related molecular functions, such as protein heterodimerization activity, molecular function regulator, and DNA-binding transcription factor activity. Details of all enriched GO categories at 3 and 6 hpi are provided in Supplementary Tables S7 and S8.

### 3.3. Pathway Analysis of DEGs Based on KEGG

**Figure 3:**
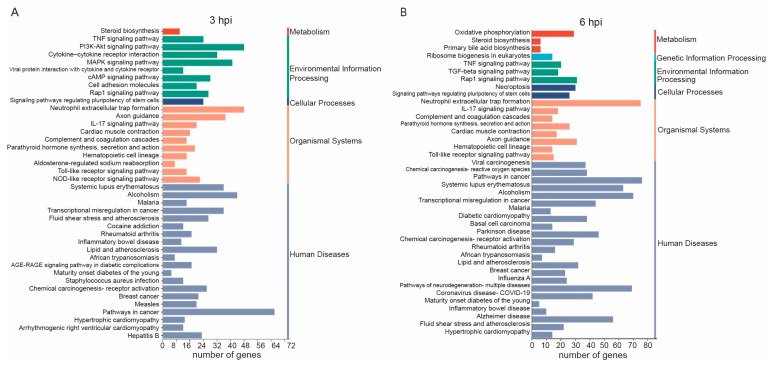
KEGG analysis of genes identified in each group at 3 and 6 hpi. (**A**) KEGG analysis of DEGs at 3 hpi. (**B**) KEGG analysis of DEGs at 6 hpi.

The KEGG database classifies orthologous genes based on pathways, offering valuable insights into predicting the biological processes and phenotypic traits of genes [25]. The KEGG database was utilized to map DEGs to reference signaling pathways in CRFK cells following FHV-1 infection. The top 40 enriched pathways were identified based on padj < 0.05, (Figure 3). At 3 and 6 hpi, DEGs were mainly related to environmental information processing, cellular processes, organismal systems, metabolism, and human diseases. Additionally, at 6 hpi, there was a correlation with genetic information processing. Given our focus on host immune responses to FHV-1 infection, identifying immune-related pathways within the host transcriptome is of particular importance.

### 3.4. DEG Heatmaps of KEGG Pathways Associated with Immunity

**Figure 4:**
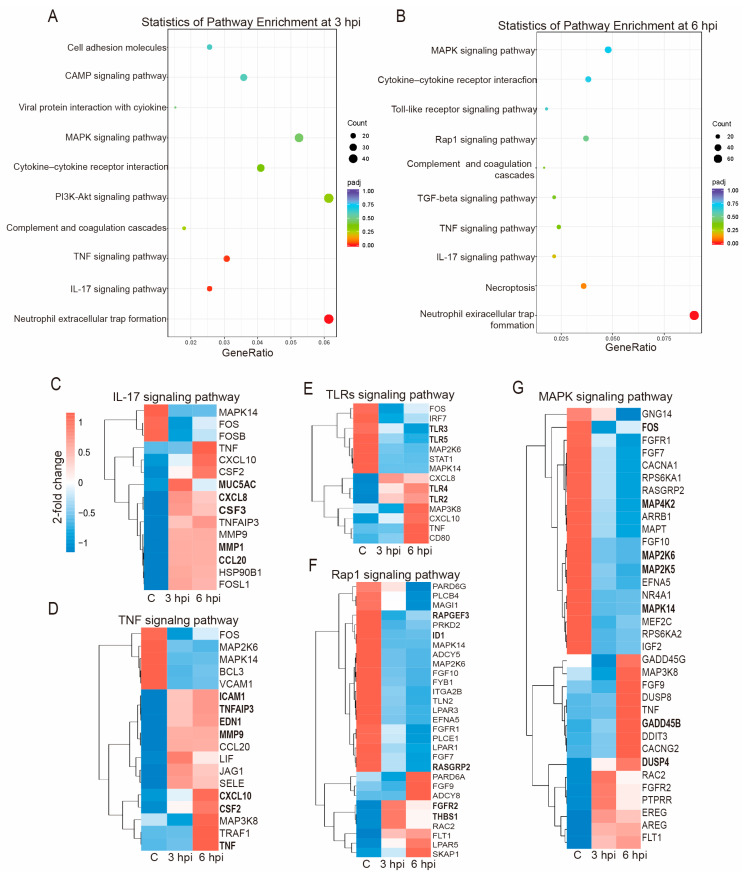
Top 10 specified KEGG pathways associated with immunity. (**A**) KEGG pathways of DEGs at 3 hpi associated with immunity. (**B**) KEGG pathways of DEGs at 6 hpi associated with immunity. (**C**) DEGs involved in the IL-17 signaling pathway and the expression level indicated in log2 fold changes. (**D**) DEGs in TNF pathway. (**E**) DEGs in TLR pathway. (**F**) DEGs in Rap1 pathway. (**G**) DEGs in MAPK pathway. DEGs validated via RT-qPCR at 6 hpi, as shown in Figure 5, are highlighted in bold.

Next, we performed KEGG pathway enrichment analyses to further define DEG functions in the early stages of FHV-1 infection. Focusing on pathways related to host immunity, the top 10 enriched immune-related pathways based on padj are listed (Figure 4). The KEGG analysis results were similar for both the 3 and 6 hpi groups. At 3 and 6 hpi, the co-enriched immune pathways potentially associated with FHV-1 infection included neutrophil extracellular trap formation, the IL-17 signaling pathway, the TNF signaling pathway, and complement and coagulation cascades. Specifically, at 3 hpi, the PI3K-Akt signaling pathway, viral protein interaction with cytokine and cytokine receptors, the cAMP signaling pathway, and cell adhesion molecules were enriched. At 6 hpi, pathways such as necroptosis, the TGF-beta signaling pathway, the Rap1 signaling pathway, and the TLRs signaling pathway were uniquely enriched. The IL-17 and TNF pathways exhibited relatively high gene ratios at 6 hpi compared to the control and 3 hpi groups. Most DEGs in these pathways were upregulated in both the 3 and 6 hpi groups, with many genes showing increased expression over time (Figure 4C,D). In contrast, the Rap1 and MAPK pathways showed a higher degree of downregulation of DEGs compared to the IL-17 and TNF pathways (Figure 4F,G). The number of downregulated DEGs in the TLRs pathway (Figure 4E) was nearly identical between the 3 and 6 hpi groups, although most DEGs exhibited greater changes at 6 hpi. These results suggest that FHV-1 infection causes significant differential expression of numerous host genes involved in multiple signaling pathways, with most DEGs related to host immunity and viral defense.

### 3.5. Validation of Immune-Related DEGs by RT-qPCR

**Figure 5:**
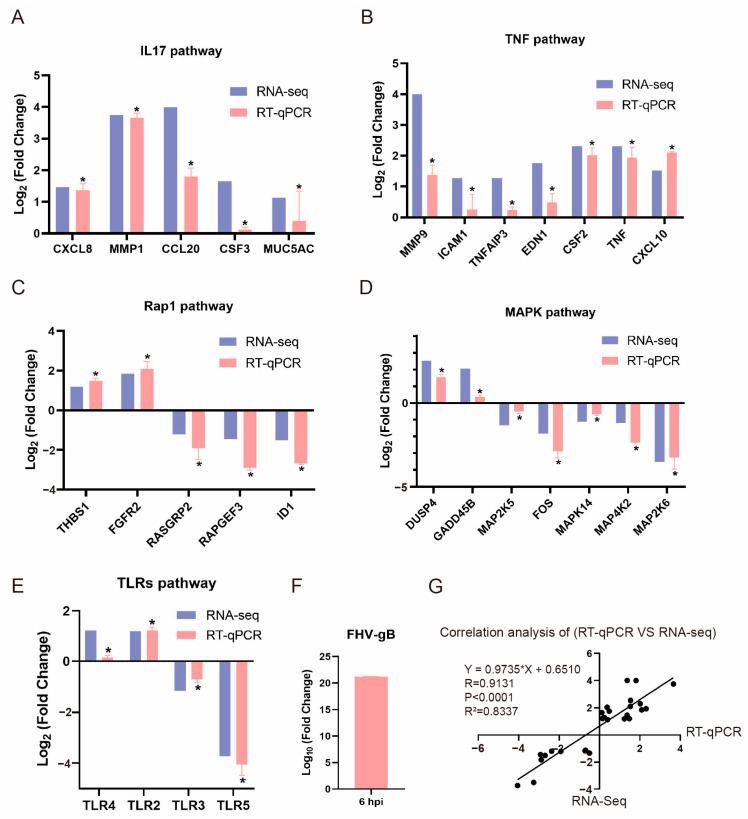
Validation of immune-related DEGs in 6 hpi by RT-qPCR. (**A**) Validation of DEGs associated with IL17 signaling pathway. (**B**) Validation of DEGs associated with TNF signaling pathway. (**C**) Validation of DEGs associated with Rap1 signaling pathway. (**C**) Validation of DEGs associated with Rap1 signaling pathway. (**D**) Validation of DEGs associated with MAPK signaling pathway. (**E**) Validation of DEGs associated with TLR signaling pathway. (**F**) FHV-1 RNA level was measured using RT-qPCR with primers specific for glycoprotein B, and the expression of each target gene was normalized to RPS7. All comparisons between the experimental conditions and the control group show statistical significance (*t*-tests, * *p* < 0.05). Three biological replicates were set up for each sample. The error bar indicates standard deviation. (**G**) Correlation of log_2_ (fold change) analyzed using data obtained from RT-qPCR (x axis) and the RNA-seq platform (y axis).

To validate the differential gene expression results from the transcriptome sequencing, we examined the expression of 28 immune-related genes at 6 hpi by RT-qPCR. These genes are primarily associated with the host’s immune defense response to FHV-1 infection, and play crucial roles in the IL-17, TNF, MAPK, Rap1, and Toll-like receptor pathways. The results show that the expression changes in the immune-related genes in RT-qPCR are consistent with those identified using RNA-seq, exhibiting a high correlation coefficient, R^2^ = 0.8337 (Figure 5). These results indicate that the reliable DEGs from RNA-seq data can serve as a valuable reference for expression profiling, with FHV-1 infection being associated with changes in DEGs that may affect DEGs and affecting the host’s immune response through signal transduction networks.

## 4. Discussion

To understand the early events of FHV-1 infection, we conducted RNA-seq analysis of FHV-1-infected CRFK cells to identify the DEGs. Our focus was on immune-related pathways, such as the IL-17, TNF, MAPK, Rap1, and TLRs pathways, and we highlighted key immune-related genes such as CXCL8, MMP1, MMP9, CSF2, CSF3, CCL20, CXCL10, TLR2, TLR3, TLR4, TNF, and FOS. These genes are likely crucial for the host’s defense against FHV-1 infection. Our findings provide potential insights into the interactions between FHV-1 and its host during the early stages of infection.

CRFK cells are commonly used as an in vitro model and are widely applied in studies of viral replication, gene expression, and antiviral drug screening [26]. However, it is important to note that CRFK cells are kidney-derived and may not fully reflect the physiological conditions of respiratory or ocular tissues, which serve as the primary targets of FHV-1 in vivo [1]. This limitation must be considered when interpreting the results, particularly regarding virus–host interactions in other tissue types. In this study, we primarily used CRFK cells to investigate the DEGs in the host induced by FHV-1 infection and to investigate the host’s immune response.

Cellular chromatin forms a dynamic structure that can either support viral genome organization for a successful viral life cycle or inhibit viral gene expression and replication by suppressing DNA accessibility [27]. In the present study, GO enrichment analysis revealed that most DEGs at 3 and 6 hpi of FHV-1 were associated with the host chromatin structure-related cellular components and signal transduction-related molecular functions (Figure 2). Upon infection, herpesvirus genomes transition from a non-nucleosomal to a chromatin structure. This rapid assembly and modification of nucleosomes in the early infection phase create a complex regulatory landscape that requires interactions among various chromatin modulation factors [28]. The Epstein–Barr virus (EBV) and Kaposi’s sarcoma-associated herpesvirus (KSHV) infection induce extensive changes in host chromatin organization and remodeling early on; evidence indicates that chromatin organization is crucial for these functions and serves a regulatory role in establishing and maintaining latent infections [29,30]. These insights provide significant understanding of host responses to FHV-1 infection and underscore the pivotal role of the chromatin structure in regulating host gene expression. Further investigation into the impacts of FHV-1 on host gene expression and function was carried out by analyzing the significant alterations in the signaling pathways.

TLRs serve as the first line of defense against viruses by recognizing invading pathogenicity and initiating downstream signaling [31,32,33]. During early FHV-1 infection in CRFK cells, KEGG analysis revealed that most DEGs were associated with immune responses and antiviral effects. The expressions of TLR2 and TLR4 were significantly upregulated at 3 and 6 hpi, while the TLR3 and TLR5 expressions were significantly downregulated, with more pronounced changes over time (Figure 4E). No significant change in the TLR9 expression was observed. However, in FHV-1-infected cat epithelial cells, the TLR9 expression was upregulated, the TLR3 expression was downregulated at 36 hpi, and the TLR2 expression remained unchanged [34]. These observations suggest differences in the transcriptomic changes between the early and mid-late stages of FHV-1 infection, as well as among different hosts. A similar downregulation of the TLR3 expression was observed in HSV-1-infected human corneal epithelial cells from 8 h onward [35]. A dominant-negative TLR3 allele in otherwise healthy children with HSV-1 encephalitis showed the crucial role of TLR3 in controlling HSV-1 infection [36]. This is consistent with the findings of TLR3-deficient mice infected with HSV-1 [37], HSV-2 [38], and EMCV [39] in the early stages, which showed consistently higher tissue inflammation, suggesting that TLR3 modulation may be a common immune escape mechanism for multiple herpesviruses, and TLR3 plays a key role in the antiviral response to herpesvirus infection.

TLR2 and TLR4 appear to have major roles in virus entry and innate immune recognition at the early stages of the viral life cycle [40,41,42]. In our study, TLR2 and TLR4 were upregulated at 3 and 6 hpi (Figure 4E). Previous studies have shown that TLR2 and TLR4 expressions occur as early as 1 h after HSV-1 infection [43]. The upregulation of TLR2 and TLR4 initiates the activation of MyD88 and TRIF, leading to an overproduction of inflammatory cytokines [44,45], including IL-15, TNF-α, and IFN to defend against HSV and counteract viral absorption [46,47]. Although TLR stimulation is crucial for recognizing dsRNAs and inducing type I IFNs, we did not observe significant changes in IFN expression during early FHV-1 infection, contrasting with findings in F81 cells (feline kidney cells) [48] at 24 hpi, and nasal samples from FHV-1-infected cats at 24 and 48 hpi [49]. Differences in TLR expression during FHV-1 infection in these studies may stem from variations in the infection times, host species, or viral strains used.

Viral infections can trigger immune responses, including cytokine and chemokine expression [50,51,52]. We observed a significant increase in cytokines and chemokines, including CXCL8, CSF2, CSF3, CCL17, CCL20, CXCL10, TNF, and CXCR4. Most of these factors exhibited a more pronounced upregulation at 6 hpi compared to 3 hpi (Figure 4). The IL-17 and TNF pathways are vital for the host cell defense against viral invasion, working together to clear herpesviruses by modulating immune and inflammatory responses, cell death, immune cell activity, and epithelial barrier function [51,53]. Activation of the IL-17 pathway stimulates the production of CXCL1, CXCL2, CXCL5, CXCL8, and IL-8, which aid in neutrophil recruitment, thereby enhancing local immune responses [54]. In our transcriptome data, only CXCL8 exhibited notable changes in the early phase of FHV-1 infection (Figure 4C). Previous studies have demonstrated that the expressions of CCL3, 4, 5, 7, 8, 9, 17, and 20, as well as those of CXCL1, 2, 4, and 5, play essential roles in the innate immune response against HSV [40,55,56,57]. In our study on the early infection phase of FHV-1, the upregulation of CCL17, CCL20, and CXCL10 among these cytokines and chemokines, as well as that of TNF were observed (Figure 4), while there were no significant changes in IFNs. A notable increase in TNF at 24 hpi and IFN-α at 36 hpi was observed in FHV-1-infected feline respiratory epithelial cells cultured at an air–liquid interface [34]. Additionally, nasal samples from cats positive for FHV-1 mRNA displayed significantly increased transcription of TNF and IFN-γ [49]. IL-17 acts synergistically with TNF and IFN-γ to enhance the host’s immune response to herpesviruses, potentially improving viral clearance in the short term but resulting in severe tissue damage and immunopathology in the long term [58]. These findings highlight the importance of early therapeutic interventions in FHV-1 infection to prevent excessive immune response.

The host’s immune system is activated to combat the pathogen in the early or mid-stages of infection [59,60]. DNA viruses can manipulate host MAPK pathways to promote viral internalization, dysregulate the cell cycle, and regulate viral replication [61,62]. Our early transcriptome analysis shows the downregulation of FOS in the MAPK pathway (Figure 5G). Previous studies identified c-Fos as a host factor involved in KSHV propagation, KSHV infection leads to accumulation of c-Fos [63], and silencing c-Fos expression reduces KSHV propagation [64]. Multiple viruses, including HSV-1, and severe acute respiratory syndrome coronavirus 2 (SARS-CoV-2), hijack p38 MAPK activation to facilitate viral replication [62]. Dysregulation of c-Fos is also common in hepatocellular carcinoma [65]. In transneuronal infection, HSV-2 induces FOS expression in spinal neurons by the fourth day [66]. The levels of activated MAPK, and the expression and stabilization of c-Fos were significantly increased in cells infected with HSV-2 from 2 to 16 hpi [67]. The transcription of Fos was activated by the Varicella-zoster virus (VZV) from 12 to 48 hpi [68]. In our transcriptome data of FHV-1 infection, we observed that FOS was downregulated by 3.0 and 1.8 times at 3 and 6 hpi, respectively (Figure 5G, Supplementary Tables S3 and S4.), while at 24 hpi, it was upregulated by 1.5 times (Supplementary Table S9). We reasonably speculate that this may be due to the host initiating an immune defense early in FHV-1 infection (3 and 6 hpi) by downregulating FOS in an attempt to reduce viral propagation, thereby preventing and clearing the invading pathogens. In the later stages of infection (24 hpi), FHV-1 hijacks host MAPK pathways to promote infection by upregulating FOS. Therefore, the upregulation of FOS and MAPK activation may be key events in FHV-1 infection, highlighting its potential as a target for antiviral treatment development [69].

Although our RNA-seq analysis provides valuable insights into the differential gene expression patterns associated with FHV-1 infection, the lack of functional assays represents a significant limitation of this study. Functional assays, such as measuring cytokine production or assessing the activation of specific signaling pathways, are crucial for validating the biological relevance of the DEGs identified in our analysis. Without these assays, it becomes challenging to establish direct causal relationships between changes in gene expression and the immune response to FHV-1. Future research should aim to incorporate these functional assays to strengthen our findings and provide a more comprehensive understanding of the FHV-1 and host interactions.

## 5. Conclusions

In conclusion, this study presents the first systematic transcriptome analysis of CRFK cells during the early stages of FHV-1 infection using RNA-seq. Our results demonstrate alterations in the host transcriptome during FHV-1 infection, indicating that FHV-1 triggers immune responses in CRFK cells and impacts early defense mechanisms. These findings contribute to our understanding of FHV-1–host interactions and lay the groundwork for developing strategies to prevent and treat FHV-1 infection.

## Data Availability

All data are presented in the article, and the original data can be obtained by email asking the author.

## Supplementary Materials

The following supporting information can be downloaded at: https://www.mdpi.com/article/10.3390/vetsci11110529/s1, Figure S1: Expression of housekeeping genes; Table S1: Primers used for RT-qPCR assays; Table S2: Number of reads of all bases detected using RNA-seq in FHV-1-infected and control CRFK cells; Table S3: Differentially expressed genes in the uninfected and 3 hpi CRFK cells; Table S4: Differentially expressed genes in the uninfected and 6 hpi CRFK cells; Table S5: Genes expressed only in the CRFK cells at 3 hpi; Table S6: Genes expressed only in the CRFK cells at 6 hpi; Table S7: GO annotation information of genes in the CRFK cells at 3 hpi; Table S8: GO annotation information of genes in the CRFK cells at 6 hpi; Table S9: Differentially expressed genes in the uninfected and 24 hpi CRFK cells.
