# Supplementary material for: Early Transcriptional Changes in Feline Herpesvirus-1-Infected Crandell-Rees Feline Kidney Cells
Source: Vet Sci. 2024 Oct 30;11(11):529. doi: 10.3390/vetsci11110529 (PMC11599068; doi:10.3390/vetsci11110529)
Supplement: Supplementary file 1 [file vetsci-11-00529-s001.zip › Supplementary files/Supplementary Figure S1-Expression of housekeeping genes.pdf]

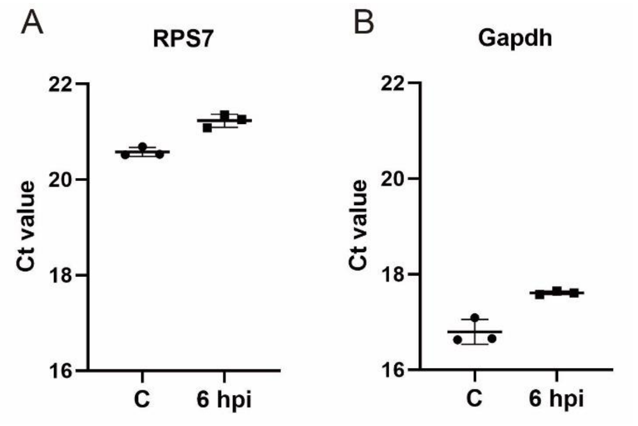

Figure S1. Expression of housekeeping genes. Expression levels of RPS7 (A) were relatively more similar under different conditions, outperforming GAPDH (B).
