# Supplementary material for: Early Transcriptional Changes in Feline Herpesvirus-1-Infected Crandell-Rees Feline Kidney Cells
Source: Vet Sci. 2024 Oct 30;11(11):529. doi: 10.3390/vetsci11110529 (PMC11599068; doi:10.3390/vetsci11110529)
Supplement: Supplementary file 1 [file vetsci-11-00529-s001.zip › Supplementary files/Supplementary Table S1-Primers used for RT-qPCR assays.pdf]

Table S1. 28 immune related genes were selected for confirmation. The primers used for qRT-PCR assays are listed.

Target gene	Sequence (5′-3′)
CXCL8-F	TCGATGCCAGTGCATAAAAACT
CXCL8-R	GTGTGGGCCACTGTCAATCA
MMP1-R	TTCGGGGAGAAGTGATGTTC
MMP1-F	CAAGTCCATTTGGCAGGTTT
MUC5AC-F	AAAGACACCAGTAGTCACTCAGCAA
MUC5AC-R	CTGGGAAGTCAGTGTCAAACCA
MMP9-F	TGCCACTTCCCTTTCACCTTC
MMP9-R	TTGCCTTCTCCATTGCCGTC
ICAM1-F	CTCACGGTGGTGCTGCTC
ICAM1-R	GTGCGGCACGAGAAATTG
CSF2-F	TC GAATTCATGTGGCTGCAGAACCTGC
CSF2-R	GCATTTCGGCTCCTCAGGGTCAAACAT
THBS1-F	AGCATCCGCAAAGTGACTGA
THBS1-R	CTCCGTTGTGGTAGCAGAG
TLR3-F	AAACTGTTGGAGGGTCTTGAG
TLR3-R	AGAATGTGGAGGTGAGAAAGAC
TLR5-F	TTCCTTCCGCCAGGAGTATTTAGC
TLR5-R	GGAGTTCGCACTCACAGATGAACT
TLR4-F	GCTGGCAATTCTTTCCAGGACAAC
TLR4-R	TCTGGAGGGAGTGAAGAGGTTCAT
TLR2-F	AGACTCTACCAGATGCCTCCTTCT
TLR2-R	GCGTGAAAGACAGGAATTCACAGG
CSF3-F	TGGACATCACCGACTTTGCT
CSF3-R	GAAGGCCGAGGTGAAGGTTG
CCL20-F	AAGTCAGAAGCAGCAAGCAG
CCL20-R	CAGCCTGTTTCACCCAGTTCT
CXCL10-F	TGCCATCATTTCCCTACATTCTT
CXCL10-R	CAGTGGTTGGTCACCTTTTAGGA
TNF-F	CACATGGCCTGCAACTAATC
TNF-R	AGCTTCGGGGTTTGCTACTAC
TNFAIP3-F	GCCTCCTGCAAGAACATCCT
TNFAIP3-R	TAGCCGTTACACTTGGCGTT
EDN1-F	AGCTCAGGGGTCAAGACAGT
EDN1-R	ACGGCGTCCAACCTTCTTATT
GADD45B-F	CTTTTGCTGCGACAACGACA
GADD45B-R	TGGTTGTTTCCTCGGCTCTC
MAP2K5-F	AGCTTGCTCTTGGGAGGTTT
MAP2K5-R	GGTTGTTTTCGCATGCTCATC
FOS-F	TTATCCCAACGGTGACTGCC
FOS-R	TCCTTTCCCTTCGGATTCTCC
MAPK14-F	AGATTATGCGTCTGACGGGG

MAPK14-R	GTACTGGGCAAAGTAGGCGT
MAP4K2-F	CTGCAGCACCCATTCACAAC
MAP4K2-R	AGCCTCGAGATCACAGTCCT
DUSP4-F	CATAGTCTACGACGAGCGCA
DUSP4-R	AGGGCCTTCGTTTTGGAACA
MAP2K6-F	AGTTGGCGATCCTTCGGTTT
MAP2K6-R	TACGTTGGCCGTTCTTTGGA
ID1-F	CCAGCACGTCATCGACTACA
ID1-R	CAAAATGCGATCGTCCGCTG
FGFR2-F	TCACTTTGGGAGGTTCACCC
FGFR2-R	TTCATTGGTGCAGTTTGCCG
RASGRP2-F	AGCTCGCTGAGCAGATCAAG
RASGRP2-R	GGATCTTGCAGAAGGAGCGA
RAPGEF3-F	AACCTGGAGCGTTTCATGCG
RAPGEF3	GCCAGCTTGATGAACTTCCTG
FHV-gB-F	AGAGGCTAACGGACCATCGA
FHV-gB-R	GCCCGTGGTGGCTCTAAAC
RPS7-F	GTCCCAGAAGCCGCACTTTGAC
RPS7-R	CTCTTGCCCACAATCTCGCTCG
GAPDH-F	CTCATGACCACAGTCCATGC
GAPDH-R	GTGAGCTTCCCATTCAGCTC
