# Supplementary material for: Early Transcriptional Changes in Feline Herpesvirus-1-Infected Crandell-Rees Feline Kidney Cells
Source: Vet Sci. 2024 Oct 30;11(11):529. doi: 10.3390/vetsci11110529 (PMC11599068; doi:10.3390/vetsci11110529)
Supplement: Supplementary file 1 [file vetsci-11-00529-s001.zip › Supplementary files/Supplementary Table S2-Number of reads of all bases detected using RNA-seq in FHV-1-infected and control CRFK cells.pdf]

Table S2. To guarantee ideal results for genomic mapping and differential gene change analysis, raw reads were filtered to remove low quality data with a total of 675 million (675 922 468) clean reads acquired.

Library	Number of raw reads	Number of clean reads	Number of uniquely mapped reads	Percentage of reads mapped
C-1	46309052	45046154	40062750	88.94%
C-2	48042376	46951258	41914658	89.27%
C-3	46473464	44956942	39767881	88.46%
C-4	46812932	45548676	40899686	89.79%
C-5	49100078	47941318	43036991	89.77%
3 hpi-1	49444530	48459000	41565954	85.78%
3 hpi-2	46024300	44989022	38766674	86.17%
3 hpi-3	43644294	42563756	36496597	85.75%
3 hpi-4	46524952	44899138	38404001	85.53%
3 hpi-5	42269774	39945348	34173643	85.55%
6 hpi-1	46416142	45005734	33048240	73.43%
6 hpi-2	39341612	38293042	28914048	75.51%
6 hpi-3	50401554	49274474	37013112	75.12%
6 hpi-4	45760342	44848910	33667395	75.07%
6 hpi-5	48247832	47199696	34680460	73.48%
Total	694813234	675922468	562412090	
